# Mendelian Randomization Analysis of Non-small Cell Lung Cancer and Five Exposure Factors Based on Pathway Enrichment Analysis

**DOI:** 10.7150/jca.98769

**Published:** 2024-10-28

**Authors:** Zishun Guo, Jieshu Zhang, Zhuozheng Hu, Jiajun Wu, Weijun Zhou, Wenxiong Zhang, Shuqiang Zhu

**Affiliations:** 1 Department of Thoracic Surgery, The Second Affiliated Hospital, Jiangxi Medical College, Nanchang University, Nanchang, China, 330006.; 2 Department of Gastroenterology, Traditional Chinese Medicine Hospital of Wannian, Wannian, China, 335599.; 3 Department of Cardiovascular Surgery, The Second Affiliated Hospital, Jiangxi Medical College, Nanchang University, Nanchang, China, 330006.

**Keywords:** Mendelian randomization analysis, Non-small cell lung cancer, Exposure factors, Pathway enrichment analysis

## Abstract

**Background:** Increasing knowledge has made it crucial to identify and minimize potential risk factors in order to prevent non-small cell lung cancer (NSCLC). This initiative aims to utilize Mendelian randomization analysis to identify exposure factors that could be causally linked to NSCLC. The results will help create new ways of controlling and preventing NSCLC.

**Methods:** The GEO database's NSCLC data were used to find differentially expressed genes, which were further analyzed for GO and KEGG pathway enrichment. Use pathway enrichment analysis as a guide to screen exposure factors. The exposure variables that are causally associated with NSCLC were screened using a two-sample Mendelian randomization technique. Heterogeneity and pleiotropy analyze were employed to assess the validity of the study's findings.

**Results:** Coronary atherosclerosis, cell adhesion molecule 3 (a molecule that maintains the normal structure and function of the lungs), dipeptidase 1 (one of the major adhesion receptors for neutrophils), thimet oligopeptidase (involved in hydrolyzing a variety of vasoactive signal peptides), and dipeptidyl peptidase 2 (an intracellular protease involved in the cell differentiation process and preventing cell death). The above five exposure factors were discovered to have an inverse relationship with NSCLC. Essentially, this implies that higher levels of these components can decrease the likelihood of developing lung cancer. No heterogeneity or pleiotropy was detected, and the study results were reliable.

**Conclusion:** The study identified five potential exposure variables for NSCLC, laying the groundwork for treatment and prevention strategies and suggesting a new path for future research.

## Introduction

Non-small cell lung cancer (NSCLC) is the predominant form of lung cancer, accounting for around 80% to 85% of all occurrences. Its increasing morbidity and mortality have brought a certain burden to the world 1. With growing awareness, it is crucial to pinpoint potential risk factors for lung cancer and implement enhanced strategies for early prevention of the illness. For example, smoking is a prominent risk factor for the development and occurrence of lung cancer. Avoiding smoking or stopping smoking at the earliest opportunity can greatly reduce the occurrence of lung cancer and enhance the outlook for individuals diagnosed with lung cancer 2, 3.

Pathway enrichment analysis, as a bioinformatics tool, is a powerful method used to uncover the mechanisms behind the occurrence and progression of diseases. This method analyzes sets of genes in genomic data linked to certain biological pathways to get insights into the molecular processes of disease 4.

Mendelian randomization (MR) is a novel epidemiological approach that efficiently reduces bias and provides advantages in terms of time and cost. It can accurately assess the cause-and-effect relationship between exposure variables and lung cancer outcomes while preventing any bias induced by reverse causality or confounding factors 5.

The study aims to utilize pathway enrichment analysis to thoroughly investigate the biological pathways associated with NSCLC in order to discover relevant exposure factors. Mendelian randomization was used to confirm the causal association between the screened exposure factors and NSCLC.

## Data and Methods

### Acquisition and processing of data related to lung cancer

**Figure 1:**
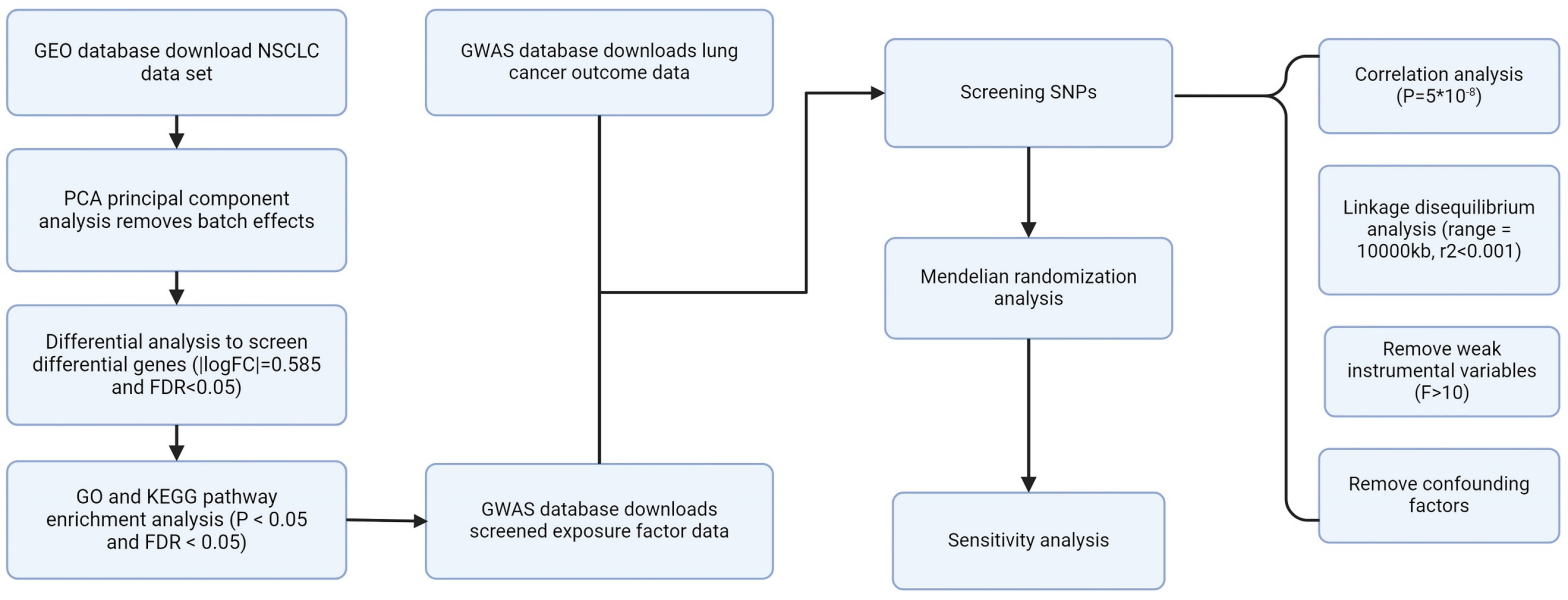
Flow chart.

The GEO website obtained four groups of NSCLC data, including GSE19188, GSE44077, GSE75037, and GSE116959. Principal Component Analysis (PCA) was used to perform batch correction on the four acquired data sets to eliminate batch effects. Subsequently, differential analysis was employed to identify genes that were expressed differently in NSCLC (**Table S1**). The screening conditions were |logFC|= 0.585 and FDR<0.05. The screening process was completed through the "limma" R code package. **Figure 1** shows our research process.

### Performing GO and KEGG pathway enrichment analysis

Kyoto Encyclopedia of Genes and Genomes (KEGG) and Gene Ontology (GO) pathway enrichment analysis was conducted utilizing genes that were expressed differently. Pathway analysis was conducted utilizing the "ClusterProfiler" R tools (P < 0.05 and FDR < 0.05) 6.

### Mendelian randomization data sources

The lung cancer outcome data required for Mendelian randomization (MR) and the exposure factor data screened for pathway enrichment analysis originate from genome-wide association research 7, 8. **Table S2** fully presents the exposure data information used in the study.

### Mendelian randomization analysis

**Figure 2:**
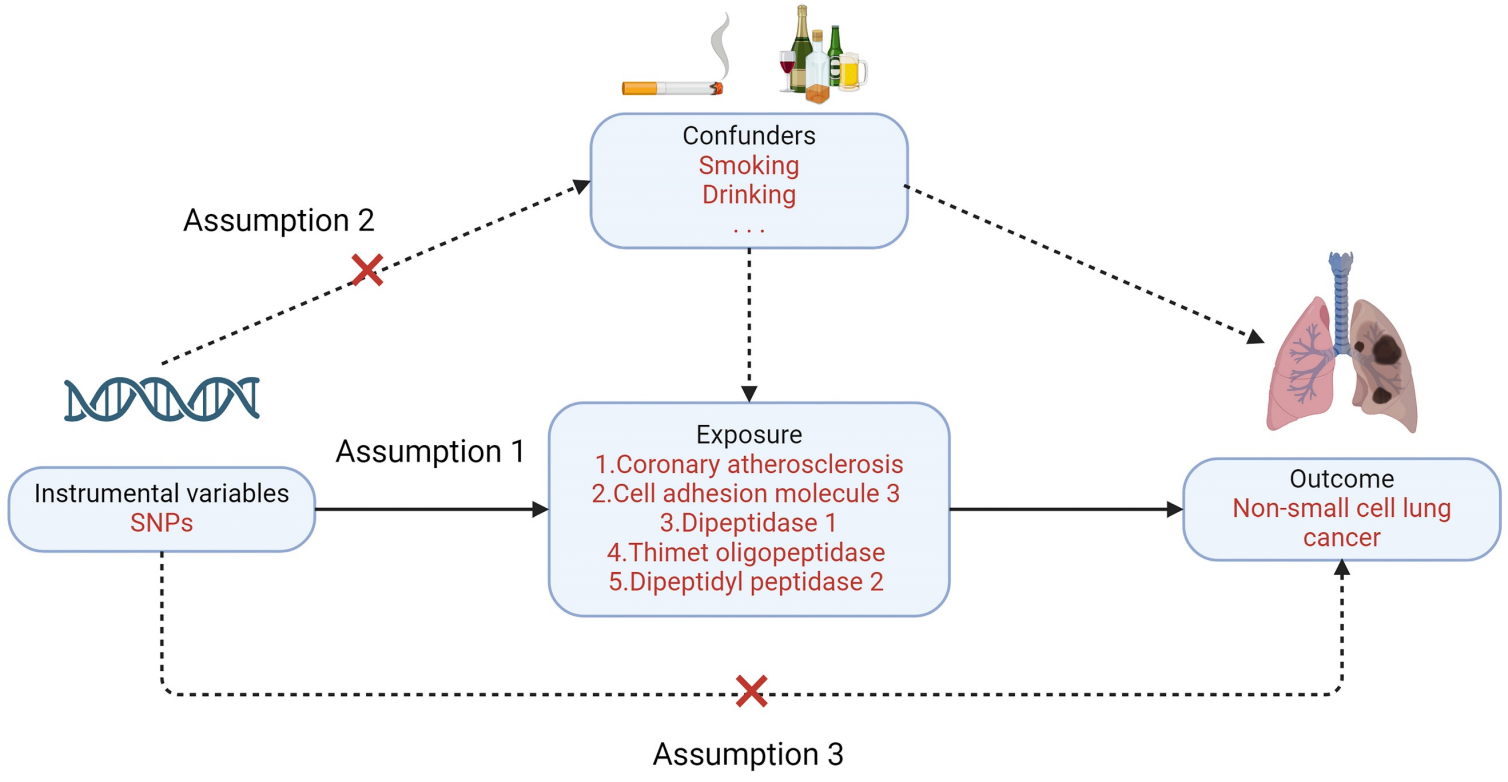
Three major assumptions of Mendelian randomization.

Mendelian randomization must satisfy three primary assumptions: 1. Exposure factors and instrumental variables have a significant association. 2. Confounding variables and instrumental variables are unrelated to one another; 3. Instrumental variables do not directly affect the results but affect the results through correlation with the degree of exposure. In this study, NSCLC was the outcome variable (Figure 2). Enrichment analysis is used to guide the screening of lung cancer-related exposure factors, and single nucleotide polymorphisms (SNPs) related to exposure factors are instrumental variables (IV) 9.

In the association analysis, we initially used a significance threshold of P<5×10^-8^ to identify SNPs that had a stronger correlation with the exposure component. Subsequently, to ensure the independence of the instrumental variables, we performed a linkage disequilibrium (LD) analysis according to the criteria of a 10,000 kb range and a r2 value below 0.001 10. Afterwards, the F test was used to exclude factor variables that were considered to be weak, using a standard threshold of F >10 11. Five approaches were employed to establish the causative association between the exposure variables and NSCLC outcomes. These methods include the inverse variance weighted (IVW) method, simple mode, weighted median, MR Egger, and weighted mode 12. The IVW method is widely acknowledged as the primary approach for establishing causal linkages because of its strong analytical results and efficient handling of measurement mistakes 13. To ensure the rigor of the study, we calculated the IVW and MR Egger's Cochran Q statistics to assess the heterogeneity among different SNPs, and the MR-Egger intercept test tested the pleiotropy of the study results. The test standards for heterogeneity and pleiotropy were P>0.05 14. Ultimately, a leave-one-out technique was used to evaluate the influence of individual SNPs on the causal relationship between exposure variables and NSCLC outcomes. The experiments were conducted using R software (version 4.2.2). The code package used for analysis is the "TwoSampleMR" package. The screening criteria used for all methodologies are shown in Table S3.

## Results

### Data processing and pathway enrichment analysis

We conduct PCA (principal component analysis) on the data to remove batch effects across various data sets. The visualization graphic is shown in Figure S1. Next, using differential analysis, we screened genes differentially expressed in lung cancer for further analysis. (logFC = 0.585, FDR<0.05) (**Figure S2**).

### Analysis of pathway enrichment

**Figure 3:**
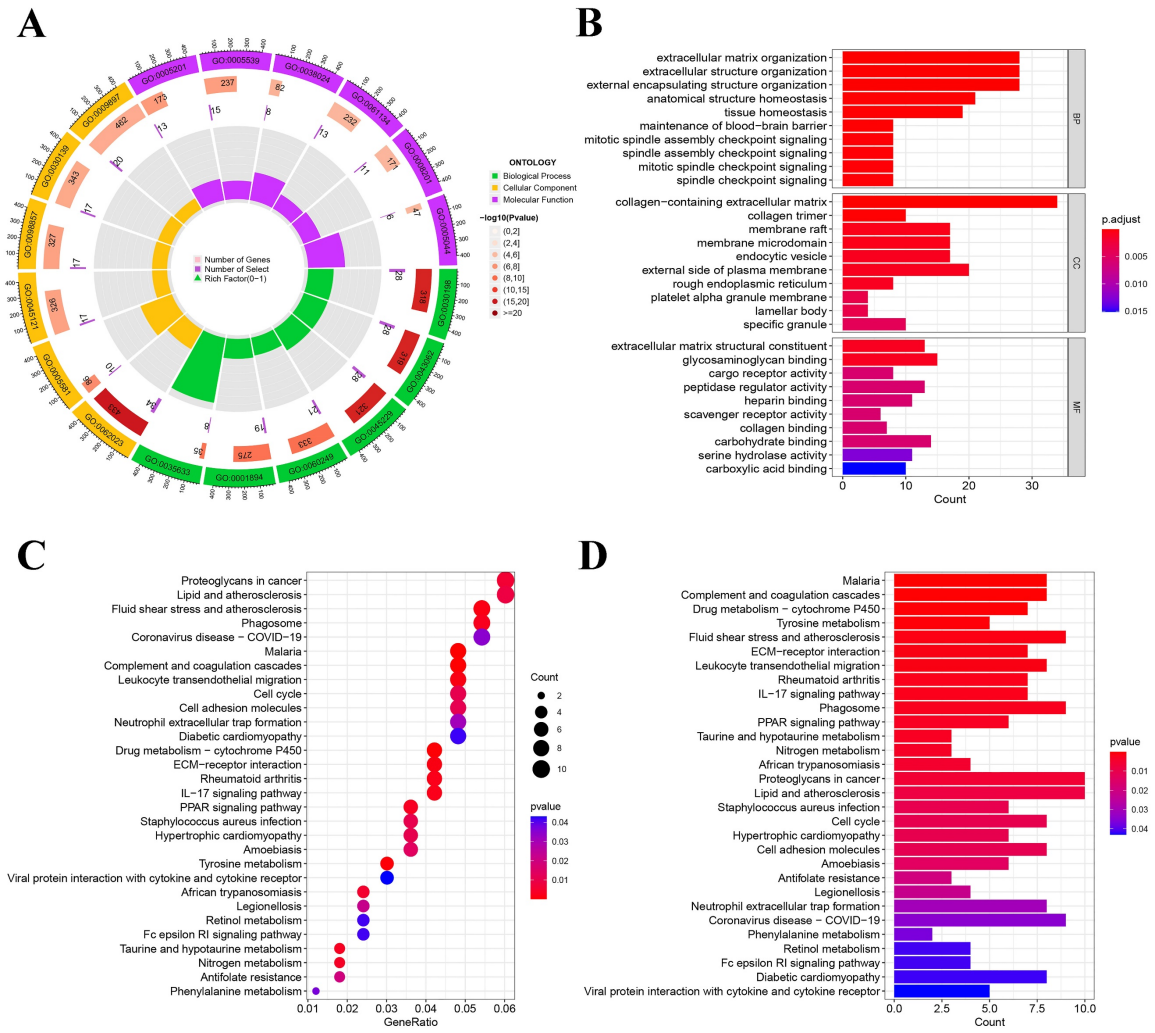
Non-small cell lung cancer pathway enrichment analysis. (A) Circle chart of GO enrichment analysis; (B) Histogram of GO enrichment analysis; (C) Bubble chart of KEGG enrichment analysis; (D) Histogram of KEGG enrichment analysis.

We conducted KEGG and GO pathway enrichment analyses on NSCLC using genes that were differentially expressed. Highly enriched pathways in GO enrichment analyses include GO:0062023 collagen-containing extracellular matrix, GO:0030198 extracellular matrix organization, and GO:0005539 glycosaminoglycan binding (**Figure 3A-B**). hsa05205: Proteoglycans in cancer, hsa05417: lipid, and atherosclerosis were highly enriched in KEGG enrichment analysis (**Figure 3C-D**). After screening and validating the entire enriched pathway, we identified several enriched pathways to guide the screening of exposure factors. For example: hsa05418: fluid shear stress and atherosclerosis; hsa05417: lipid and atherosclerosis; GO:0052547: regulation of peptidase activity; GO:0010466: negative regulation of peptidase activity; GO:0010811: positive regulation of cell-substrate adhesion, etc. We present all pathways obtained from our analysis in **Table S4** and **Table S5**.

### Acquisition of exposure factors

After screening After screening guided by pathway enrichment analysis, five exposure factors were included in the study, including coronary atherosclerosis, cell adhesion molecule 3, dipeptidase 1, thimet oligopeptidase (THOP1), and dipeptidyl peptidase 2. After quality evaluation of exposure factors, the number of SNPs screened out was 77, 17, 14, 14, and 9, respectively. We provide the SNP information for valid IVs in **Table S6**. All single nucleotide polymorphisms (SNPs) had F values exceeding 10, suggesting the effectiveness of all five instrumental variables (IVs).

### Two-sample Mendelian randomization between five exposure factors and NSCLC

**Figure 4:**
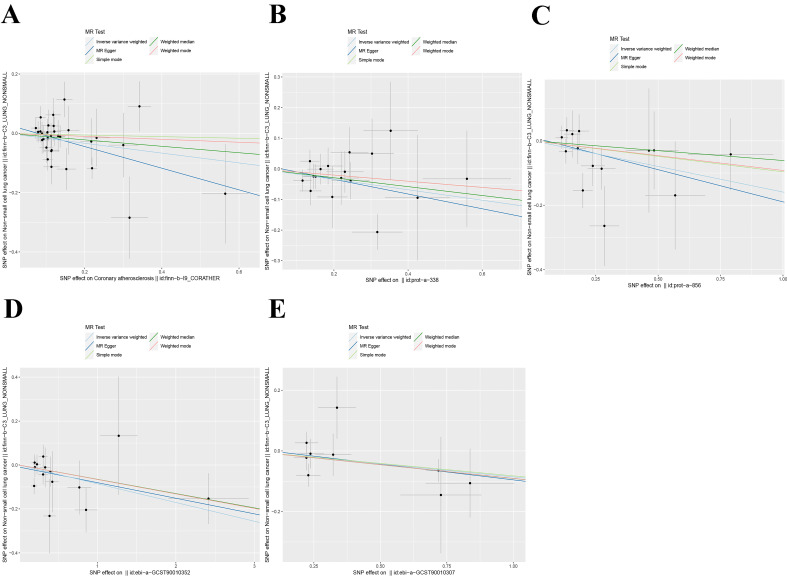
Scatter plot of Mendelian randomization analysis of five exposure factors. (A) Coronary atherosclerosis; (B) Cell adhesion molecule 3; (C) Dipeptidase 1; (D) Thimet oligopeptidase; (E) Dipeptidyl peptidase 2.

We employed five methodologies to evaluate the presence of a causal connection between the screened exposure factors and the risk of NSCLC (including MR Egger, IVW, weighted median, simple mode, and weighted mode). The IVW technique is the primary approach used in our study assessment. The findings indicate that there is an inverse causal association between the five exposure factors we examined and the prediction of NSCLC using the IVW technique (**Figure 4**). Among them, coronary atherosclerosis (OR = 0.85, 95% CI: 0.73-0.98); P = 0.02), cell adhesion molecule 3 (OR = 0.84, 95% CI: 0.73-0.97); P = 0.02), dipeptidase 1 (OR = 0.85, 95% CI: 0.74-0.99); P = 0.03), THOP1 (OR = 0.92, 95% CI: 0.86-0.98); P = 0.01), and dipeptidyl peptidase 2 (OR = 0.92, 95% CI: 0.85-1.00); P = 0.04). Consequently, elevations in these variables can diminish the likelihood of developing lung cancer. We display the specific values analyzed in the table and visualize them in **Table 1**.

### Validation of Mendelian randomization findings

**Figure 5:**
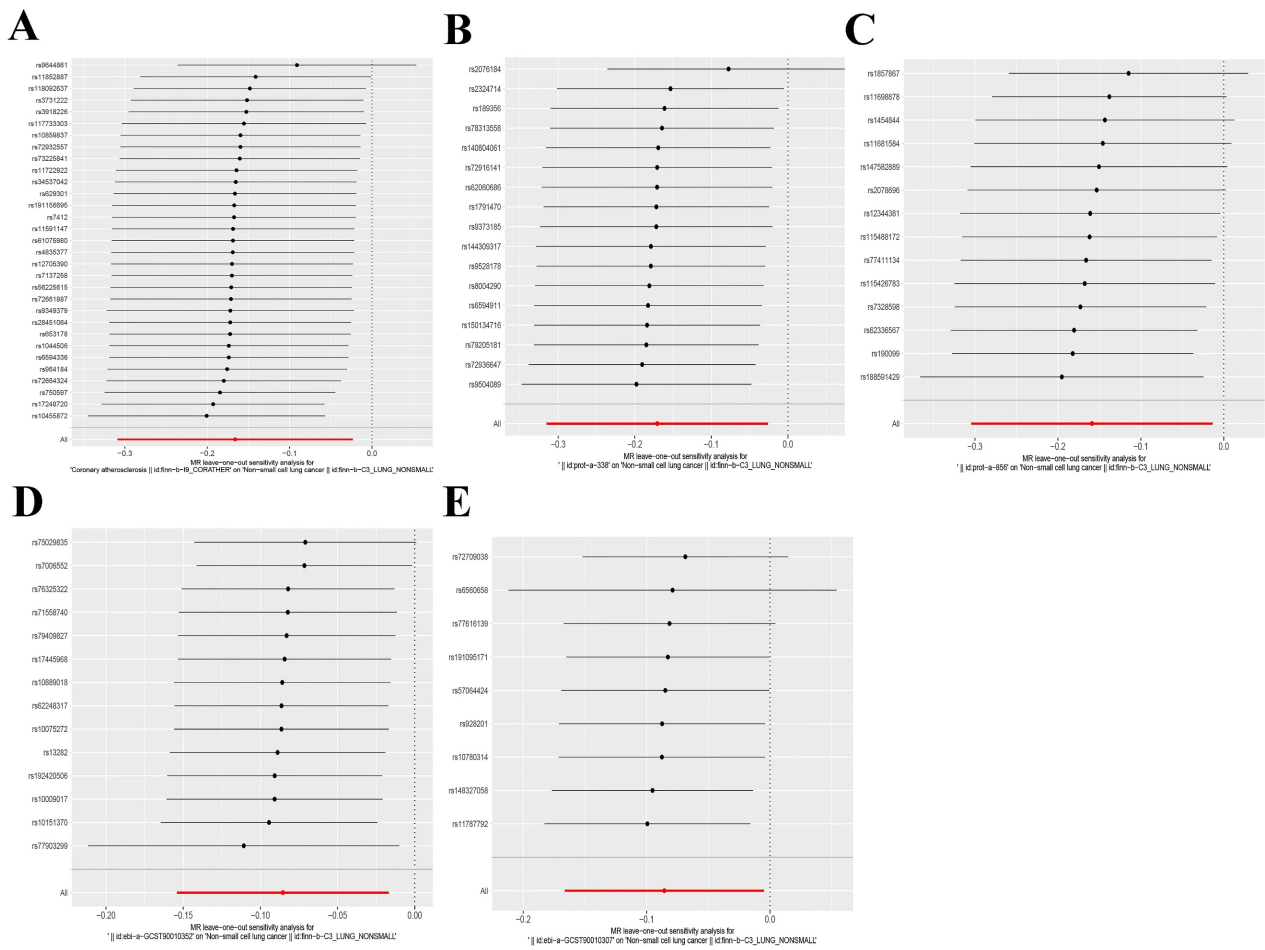
Leave-one-out analysis of five exposure factors. (A) Coronary atherosclerosis; (B) Cell adhesion molecule 3; (C) Dipeptidase 1; (D) Thimet oligopeptidase; (E) Dipeptidyl peptidase 2.

**Figure 6:**
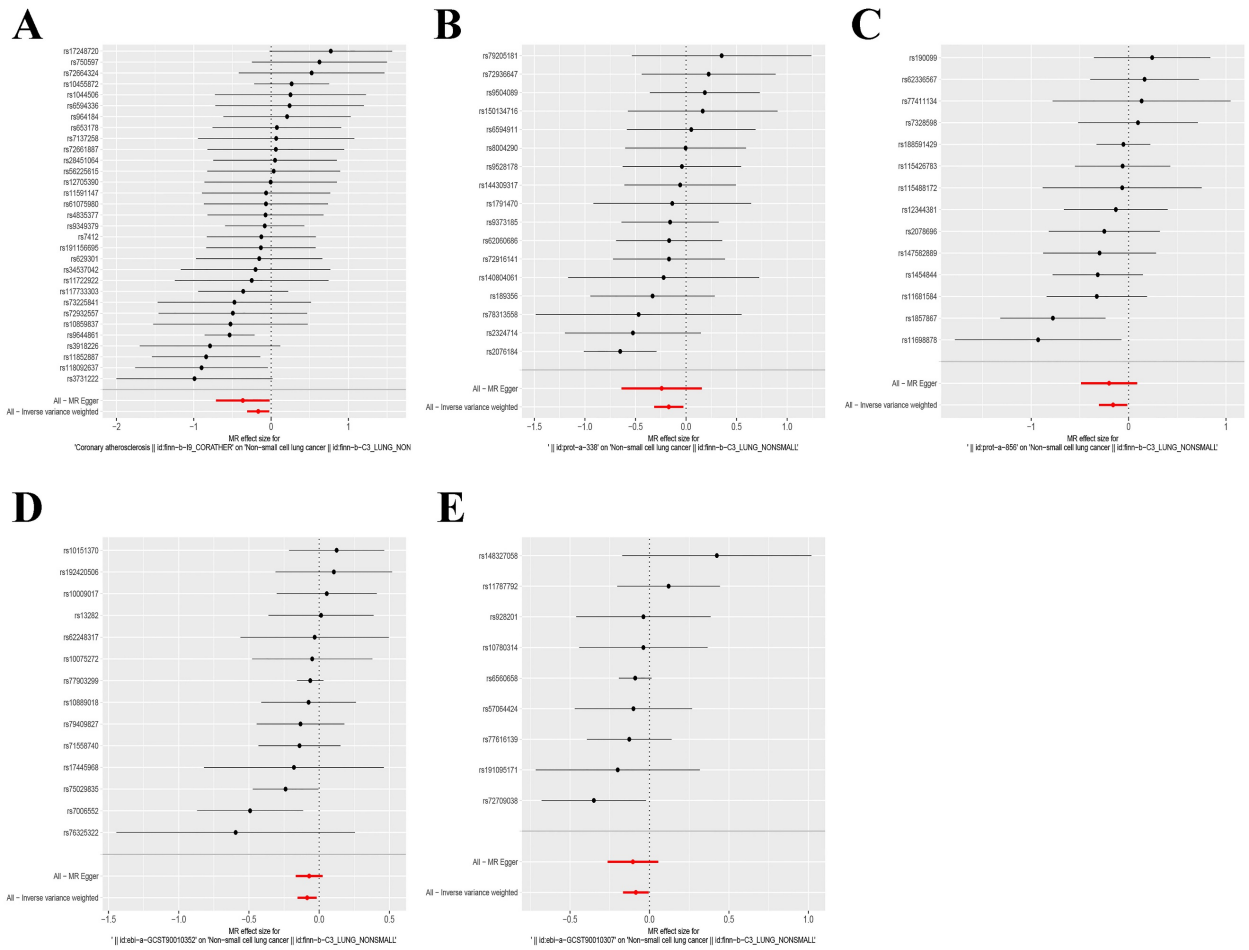
Forest plot of SNPs for five exposure factors. (A) Coronary atherosclerosis; (B) Cell adhesion molecule 3; (C) Dipeptidase 1; (D) Thimet oligopeptidase; (E) Dipeptidyl peptidase 2.

The IVW and MR-Egger approaches demonstrated the absence of heterogeneity in the five examined exposure factors. (P>0.05). In addition, the MR-Egger regression analysis demonstrated that there was no horizontal pleiotropy among the SNPs of all exposure factors. **Table 2** displays the findings from the analyses of heterogeneity and pleiotropy. Leave-one-out sensitivity analysis revealed no SNPs that significantly affected causality (**Figure 5**). Figure 6 displays the forest plot of SNPs in the MR study., and the funnel plot can be seen in **Figure S3**. The above results show that the results obtained by IVW are reliable.

### Verification of results

To verify the analytic results of coronary atherosclerosis, we utilised distinct exposure data (GWAS ID: ukb-d-I9_CORATHER). The IVW analysis approach confirms a significant inverse causal association between coronary atherosclerosis and NSCLC (odds ratio = 0.01, 95% confidence interval: 0.001-0.43) (**Table S7-S8**). Unfortunately, the validity of the remaining four exposure factors could not be confirmed due to the unavailability of relevant external data. The visualisation of the verification results is depicted in **Figure S4**.

## Discussion

During the previous few years, with the continuous advancement of science and technology, the examination and treatment methods for lung cancer have been continuously improved 15, 16. The perception of lung cancer danger among the general public is gradually rising. Identifying potential risk factors linked to the onset of lung cancer and taking steps to avoid or minimize exposure are crucial methods for preventing and managing lung cancer in the present day. Our work employed pathway enrichment analysis to direct the screening of exposure variables in order to identify additional risk factors that are strongly linked to lung cancer. A total of 5 exposure factors were screened out, including coronary atherosclerosis, cell adhesion molecule 3, dipeptidase 1, THOP1, and dipeptidyl peptidase 2. The results of Mendelian randomization revealed a causal association in the opposite direction between these five exposure factors and the occurrence of NSCLC. More precisely, these five characteristics of exposure were linked to a reduced likelihood of getting non-small cell lung cancer.

Our research discovered a correlation between coronary atherosclerosis and a decreased likelihood of developing NSCLC. This conclusion was corroborated by the verification of various exposure data. A study examining the correlation between coronary artery calcium (CAC) and different forms of cancer revealed that male patients with a CAC level of 400 or above had a heightened susceptibility to lung cancer 17. Chen *et al.*'s study also discovered that those with coronary artery disease (CAD) have a heightened susceptibility to lung cancer 18. This is contrary to our findings. Although the aforementioned investigations have partially elucidated the correlation between coronary atherosclerosis and lung cancer, they have not fully accounted for the impact of smoking and other confounding variables. This is the key reason that the above studies are contrary to ours. Researchers conducted a study on tumor necrosis factor (TNF) and discovered that higher levels of TNF were linked to an increased risk of coronary artery disease, while lower levels of TNF were connected with a decreased risk of lung cancer 19. While there is ongoing debate over the impact of TNF on lung cancer, it is generally accepted that TNF has a role in promoting the onset and progression of lung cancer 20, 21. Tumor necrosis factor (TNF) is a probable crucial element in the connection between coronary artery disease (CAD) and lung cancer. Cell adhesion molecules have been shown to be involved in the induction and maintenance of tissue differentiation, and their loss or downregulation is associated with the formation of various tumors 22. Our research suggests a correlation between cell adhesion molecule 3 and a reduced risk of developing NSCLC. A study on cell adhesion molecule 4 (CADM4) showed that CADM4 can impede the proliferation and spread of non-small cell lung cancer (NSCLC) cells by blocking the Akt signaling pathway 23. CADM3 may also decrease the likelihood of developing lung cancer in the same manner. The expression of CADM4 in gallbladder cancer has been observed to have a negative correlation with the poor prognosis of individuals with this type of cancer 24. However, there are still few studies on cell adhesion molecule 3. In our study, there was a reverse causal relationship between dipeptidase-1 (DPEP-1) and NSCLC. Dipeptidase-1 is a zinc-dependent metalloprotease shown to be a major adhesion receptor on the liver and lung endothelium, and its reduction reduces neutrophil recruitment to the lungs and liver 25. Dysregulation of DPEP-1 in a variety of cancers can have an impact on cancer progression. For example, in hepatoblastoma (HB), poor patient prognosis is associated with upregulation of DPEDP-1, while in breast cancer, DPEP-1 target genes may be a tumor suppressor gene 26, 27. Neutrophil recruitment is a crucial cellular characteristic of lung cancer, and DPEP-1 is believed to have a causal connection with lung cancer by influencing this cellular characteristic 28. THOP1 also has a reverse causal relationship with NSCLC. THOP1 is linked to a higher susceptibility to NSCLC. Studies have shown that the expression of THOP1 in NSCLC is less than that found in healthy lung tissue. Additionally, a drop in THOP1 expression is linked to a reduction in the 5-year disease-free survival and overall survival of individuals with NSCLC 29. THOP1 has the ability to break down a wide range of active peptides, with a particular emphasis on bradykinin (BK) 30. Bradykinin is a significant signaling molecule that plays a crucial role in tumor-associated blood vessel formation and the progression of tumors 31. THOP1, acting as an antagonist of BK, will likely reduce the likelihood of developing lung cancer by decreasing BK. Finally, our investigation confirmed a reverse causal connection between DPP2 and NSCLC. DPP2 is an enzyme that belongs to the serine protease family and has a crucial function in preserving cellular quiescence. It has been shown to be an important predictive factor in chronic lymphocytic leukemia (CLL). Inhibition of DPP2 can induce apoptosis in CLL cells 32, 33. However, there are currently few studies on its use in cancer, and it still has certain research potential.

We performed GO and KEGG pathway enrichment analyses to discover the pathways enriched in NSCLC. We subsequently identified and screened exposure variables associated with these pathways using the provided information. Mendelian randomization was used to investigate the causal link between exposure factors and the outcomes of NSCLC. And finally, we screened out five exposure factors, including coronary atherosclerosis, cell adhesion molecule 3, dipeptidase 1, THOP1, and dipeptidyl peptidase 2. Mendelian randomization provides evidence that these five exposure factors have a causal association in the opposite direction with NSCLC. This method effectively eliminates the impact of confounding factors, hence enhancing the accuracy of assessing causality. It provides new research directions and a basis for the prevention and treatment of NSCLC. However, our study also has certain shortcomings. First, our research relies on public databases, and the relevant results still need further experimental verification. Secondly, some of our research methods failed to produce the same results as the IVW method, but the SNPs included in the study all met the valid IV hypothesis, and the results obtained by the study did not show heterogeneity and pleiotropy, so our results are reliable.

## Conclusion

Based on GO and KEGG pathway enrichment analyses of NSCLC, we screened exposure factors related to lung cancer. Through Mendelian randomization analysis, five exposure factors were screened out, including coronary atherosclerosis, cell adhesion molecule 3, dipeptidase 1, THOP1, and dipeptidyl peptidase 2. The IVW method proves that these five exposure factors have a reverse causal relationship with NSCLC and may reduce the risk of NSCLC. Heterogeneity and pleiotropic effects analysis proved the accuracy of the study results. The specific mechanism of the interaction between exposure factors and NSCLC still requires further experimental verification.

## Supplementary Material

Supplementary figures and tables.

## Figures and Tables

**Table 1:** Mendelian randomization-evaluated exposure factors for the presence of non-small cell lung cancer.

Exposure	Method	Beta	OR (95%CI)	P value
**Coronary atherosclerosis**	MR Egger	-0.37	0.69(0.49-0.98)	0.05
	Weighted median	-0.11	0.90(0.73-1.11)	0.32
	Inverse variance weighted	-0.17	0.85(0.73-0.98)	0.02
	Simple mode	-0.03	0.97(0.65-1.46)	0.90
	Weighted mode	-0.05	0.95(0.63-1.43)	0.81
**Cell adhesion molecule 3**	MR Egger	-0.24	0.79(0.53-1.17)	0.25
	Weighted median	-0.14	0.87(0.70-1.06)	0.17
	Inverse variance weighted	-0.17	0.84(0.73-0.97)	0.02
	Simple mode	-0.10	0.90(0.63-1.29)	0.59
	Weighted mode	-0.10	0.90(0.64-1.28)	0.58
**Dipeptidase 1**	MR Egger	-0.20	0.82(0.61-1.09)	0.20
	Weighted median	-0.06	0.94(0.77-1.15)	0.55
	Inverse variance weighted	-0.16	0.85(0.74-0.99)	0.03
	Simple mode	-0.10	0.91(0.68-1.22)	0.53
	Weighted mode	-0.09	0.91(0.72-1.15)	0.45
**Thimet oligopeptidase**	MR Egger	-0.07	0.93(0.85-1.03)	0.17
	Weighted median	-0.07	0.94(0.85-1.03)	0.19
	Inverse variance weighted	-0.09	0.92(0.86-0.98)	0.01
	Simple mode	-0.07	0.94(0.79-1.10)	0.45
	Weighted mode	-0.07	0.94(0.85-1.03)	0.20
**Dipeptidyl peptidase 2**	MR Egger	-0.10	0.90(0.77-1.06)	0.24
	Weighted median	-0.09	0.91(0.83-1.01)	0.07
	Inverse variance weighted	-0.09	0.92(0.85-1.00)	0.04
	Simple mode	-0.08	0.92(0.79-1.08)	0.34
	Weighted mode	-0.09	0.91(0.83-1.00)	0.09

**Abbreviation**: P value: Probability; OR: Odds ratio; 95%CI: 95% Confidence Interval.

**Table 2:** Heterogeneity analysis and MR-Egger regression of 5 exposure factors associated with lung cancer.

Exposure	Heterogeneity P			MR-Egger regression	
	MR Egger	IVW		Intercept	Intercept P
Coronary atherosclerosis	0.2628	0.2379		0.0287	0.2269
Cell adhesion molecule 3	0.4751	0.5383		0.0142	0.7147
Dipeptidase 1	0.2973	0.3614		0.0105	0.7493
Thimet oligopeptidase	0.5252	0.5926		-0.0093	0.6835
Dipeptidyl peptidase 2	0.4097	0.5089		0.0089	0.7967

**Abbreviation**: Heterogeneity P: Heterogeneity P-value; IVW: Inverse variance weighted; Intercept P: Intercept P-value.

## Abbreviations

BK: bradykinin
CAC: coronary artery calcium
CAD: coronary artery disease
CADM4: cell adhesion molecule 4
CLL: chronic lymphocytic leukemia
DPEP-1: Dipeptidase-1
DPP2: dipeptidyl peptidase 2
GO: Gene Ontology
HB: hepatoblastoma
IV: instrumental variables
IVW: inverse variance weighting
KEGG: Kyoto Encyclopedia of Genes
LD: linkage disequilibrium
MR: Mendelian randomization
NSCLC: Non-small cell lung cancer
PCA: Principal Component Analysis
SNPs: single nucleotide polymorphisms
THOP1: Thimet oligopeptidase
TNF: tumor necrosis factor
