# Supplementary material for: Mendelian Randomization Analysis of Non-small Cell Lung Cancer and Five Exposure Factors Based on Pathway Enrichment Analysis
Source: J Cancer. 2024 Oct 28;15(20):6658–67. doi: 10.7150/jca.98769 (PMC11632983; doi:10.7150/jca.98769)
Supplement: Supplementary file 1 — Supplementary figures and tables. [file jcav15p6658s1.pdf]

Supplementary Material

Figure S1 PCA principal component analysis. (A) Before the batch effect is eliminated; (B) After the batch effect is eliminated.

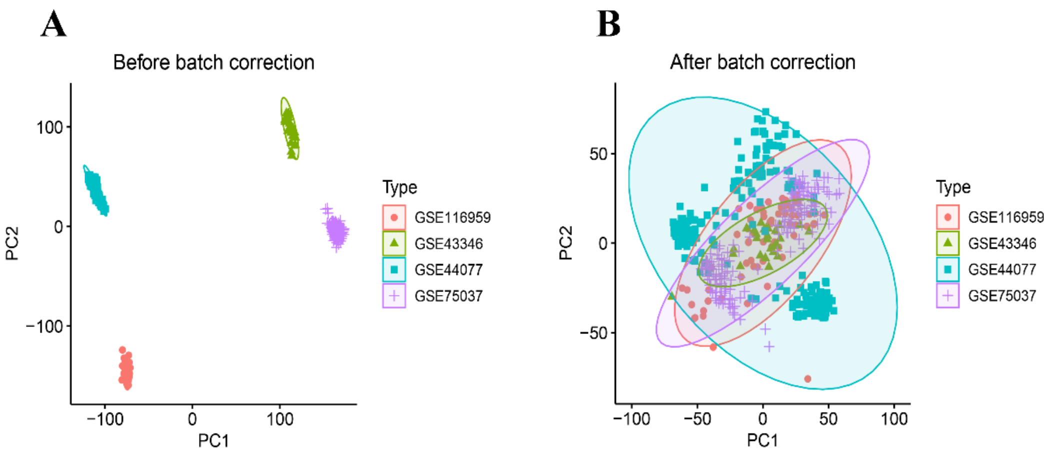

Figure S2 Screening differential genes. (A) Difference analysis volcano plot; (B) Difference analysis heat map.

A

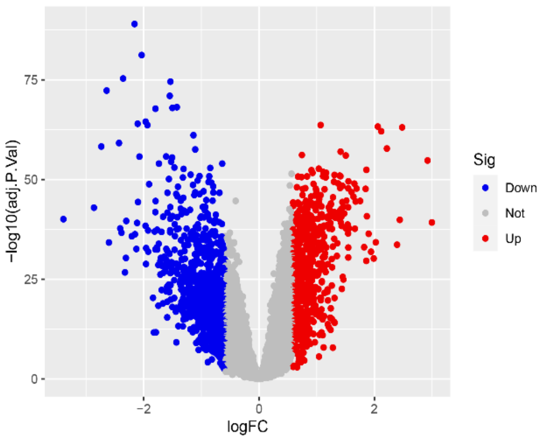

B

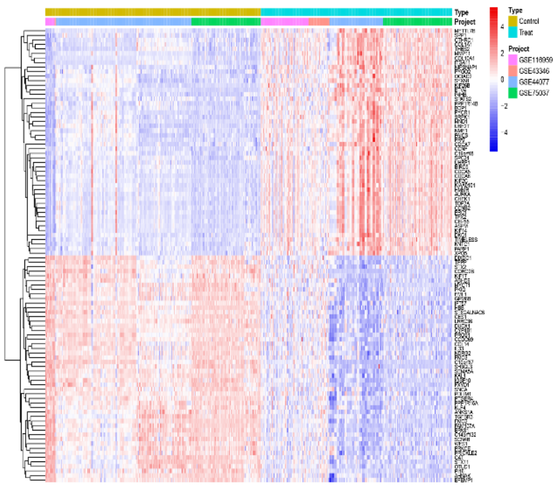

Figure S3 Funnel plot of Mendelian randomization analysis for five exposure factors. (A) Coronary atherosclerosis; (B) Cell adhesion molecule 3; (C) Dipeptidase 1; (D) Thimet oligopeptidase; (E) Dipeptidyl peptidase.

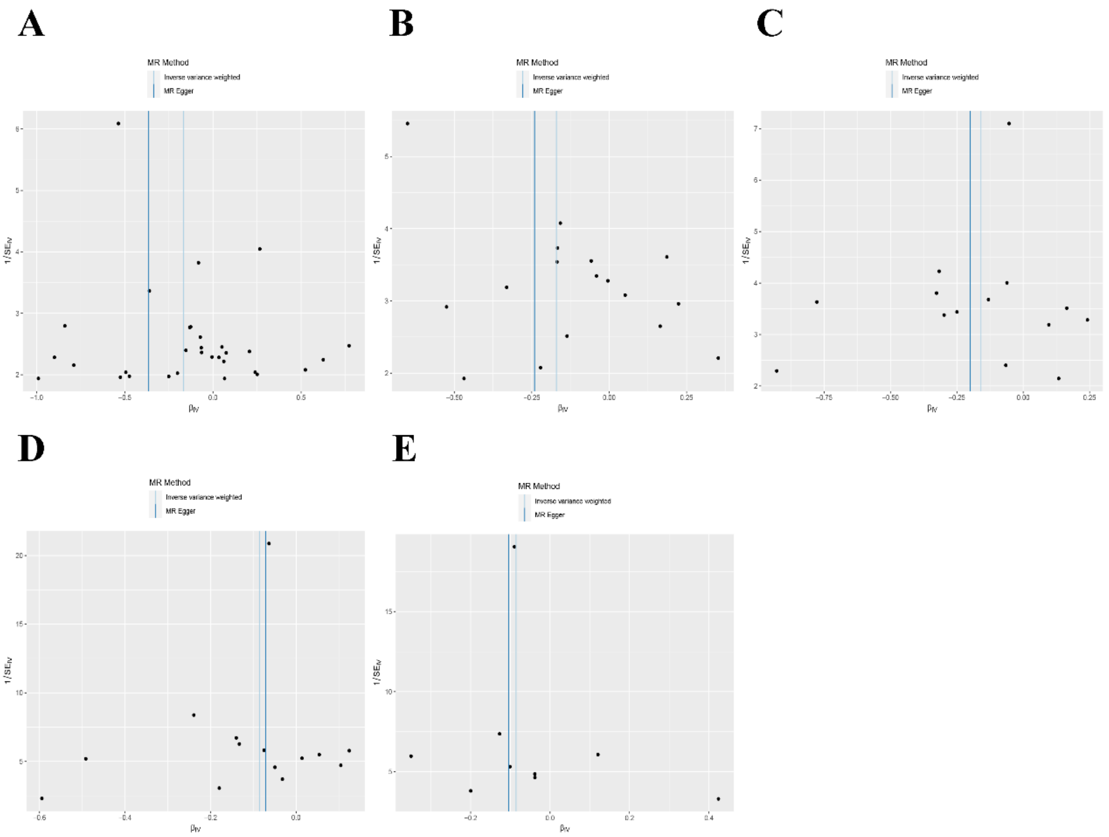

Figure S4 External data validation of coronary atherosclerosis. (A) SNPs forest plot of the validation dataset; (B) Leave-one-out sensitivity analysis of the validation dataset; (C) MR analysis scatter plot of the validation dataset; (D) MR analysis funnel plot of the validation dataset.

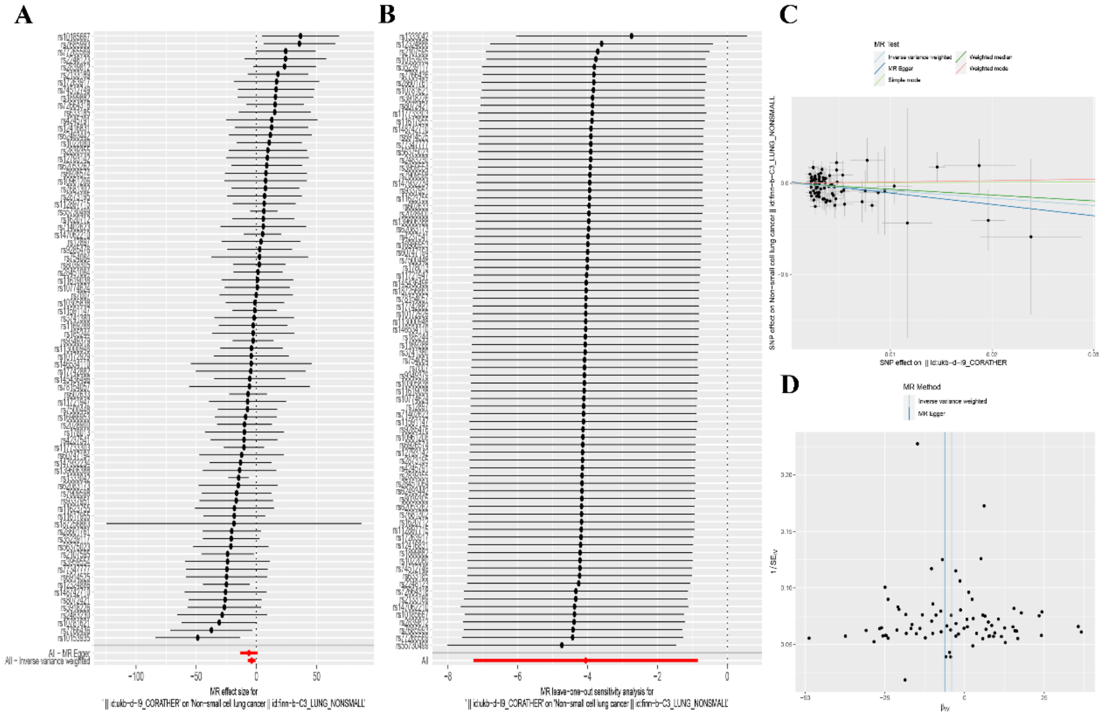

Table S1 Table 1 The top 20 differentially expressed genes obtained by differential analysis

ID	pvalue	adj.p.val
FABP4	1.43E-75	2.24E-71
TCF21	2.78E-69	2.18E-65
SH3GL3	1.99E-68	1.04E-64
ADH1B	5.84E-67	1.66E-63
AGER	6.37E-67	1.66E-63
SGCG	9.34E-67	2.09E-63
LGI3	1.00E-65	1.96E-62
FMO2	1.03E-64	1.73E-61
ITLN2	1.11E-64	1.73E-61
CA4	3.09E-63	4.39E-60
CAV1	9.13E-63	1.19E-59
CDO1	3.74E-62	4.50E-59
C10orf67	5.88E-62	6.57E-59
STX11	1.05E-61	1.10E-58
PLAC9	9.65E-61	9.44E-58
ACADL	5.83E-60	5.37E-57
SPOCK2	7.40E-60	6.44E-57
FAM107A	1.35E-59	1.11E-56

Abbreviation: P-value: Probability; adj.p.val: Adjusted P-value.

Table S2 Outcome data obtained from GWAS database.

Trait	Consortium	Sample size	Number of SNPs	GWAS ID
Non-small cell lung cancer	NA	218,792	16,380,466	finn-b-C3_LUNG_NONSMALL
Non-small cell lung cancer (all cancers excluded)	NA	175,633	16,380,305	finn-b-C3_LUNG_NONSMALL_EXALLC
Non-small cell lung cancer, LUAD	NA	218,792	16,380,466	finn-b-C3_NSCLC_ADENO
Non-small cell lung cancer, LUSC	NA	218,792	16,380,466	finn-b-C3_NSCLC_SQUAM

Abbreviation: NA: Not described.

Table S3 Summary of Methodological Screening Criteria.

Methodology	Screening Criteria
Difference analysis	$\|logFC\|= 0.585$, FDR < 0.05
GO and KEGG pathway enrichment analysis	P < 0.05, FDR < 0.05
SNPs association analysis	$P < 5\times10^{-8}$
Linkage disequilibrium analysis	range = 10000kb, r2 < 0.001
F test	F > 10
Heterogeneity analysis	P > 0.05
Pleiotropy analysis	P > 0.05

Abbreviation: P: P value；FDR：false discovery rate.

Table S4 GO pathway enrichment analysis results.

ID	Description	p-value	q-value
GO:0030198	extracellular matrix organization	0.00	0.00
GO:0043062	extracellular structure organization	0.00	0.00
GO:0045229	external encapsulating structure organization	0.00	0.00
GO:0060249	anatomical structure homeostasis	0.00	0.00
GO:0001894	tissue homeostasis	0.00	0.00
GO:0035633	maintenance of blood-brain barrier	0.00	0.00
GO:0007094	mitotic spindle assembly checkpoint signaling	0.00	0.00
GO:0071173	spindle assembly checkpoint signaling	0.00	0.00
GO:0071174	mitotic spindle checkpoint signaling	0.00	0.00
GO:0031577	spindle checkpoint signaling	0.00	0.00
GO:0045841	negative regulation of mitotic metaphase/anaphase transition	0.00	0.00
GO:1902100	negative regulation of metaphase/anaphase transition of cell cycle	0.00	0.00
GO:0033046	negative regulation of sister chromatid segregation	0.00	0.00
GO:0033048	negative regulation of mitotic sister chromatid segregation	0.00	0.00
GO:2000816	negative regulation of mitotic sister chromatid separation	0.00	0.00
GO:0050678	regulation of epithelial cell proliferation	0.00	0.00
GO:0051985	negative regulation of chromosome segregation	0.00	0.00
GO:1905819	negative regulation of chromosome separation	0.00	0.00
GO:0031960	response to corticosteroid	0.00	0.00
GO:0015670	carbon dioxide transport	0.00	0.00
GO:0052547	regulation of peptidase activity	0.00	0.00
GO:0051384	response to glucocorticoid	0.00	0.00
GO:0019755	one-carbon compound transport	0.00	0.00
GO:0033047	regulation of mitotic sister chromatid segregation	0.00	0.00
GO:0045839	negative regulation of mitotic nuclear division	0.00	0.00
GO:0002237	response to molecule of bacterial origin	0.00	0.00
GO:0050673	epithelial cell proliferation	0.00	0.00
GO:0032496	response to lipopolysaccharide	0.00	0.00
GO:0022617	extracellular matrix disassembly	0.00	0.00
GO:0051784	negative regulation of nuclear division	0.00	0.00
GO:0045926	negative regulation of growth	0.00	0.00
GO:0050878	regulation of body fluid levels	0.00	0.00
GO:0006959	humoral immune response	0.00	0.00
GO:0030071	regulation of mitotic metaphase/anaphase transition	0.00	0.00
GO:0031589	cell-substrate adhesion	0.00	0.00
GO:0051310	metaphase plate congression	0.00	0.00
GO:0007088	regulation of mitotic nuclear division	0.00	0.00
GO:0072593	reactive oxygen species metabolic process	0.00	0.00
GO:0007091	metaphase/anaphase transition of mitotic cell cycle	0.00	0.00
GO:1902099	regulation of metaphase/anaphase transition of cell cycle	0.00	0.00
GO:0051315	attachment of mitotic spindle microtubules to kinetochore	0.00	0.00

GO:0090231	regulation of spindle checkpoint	0.00	0.00
GO:0090266	regulation of mitotic cell cycle spindle assembly checkpoint	0.00	0.00
GO:1903504	regulation of mitotic spindle checkpoint	0.00	0.00
GO:0048545	response to steroid hormone	0.00	0.00
GO:0045446	endothelial cell differentiation	0.00	0.00
GO:0050727	regulation of inflammatory response	0.00	0.00
GO:2001251	negative regulation of chromosome organization	0.00	0.00
GO:0010965	regulation of mitotic sister chromatid separation	0.00	0.00
GO:0044784	metaphase/anaphase transition of cell cycle	0.00	0.00
GO:0051987	positive regulation of attachment of spindle microtubules to kinetochore	0.00	0.00
GO:0097529	myeloid leukocyte migration	0.00	0.00
GO:0015669	gas transport	0.00	0.00
GO:0043434	response to peptide hormone	0.00	0.00
GO:0007517	muscle organ development	0.00	0.00
GO:0051306	mitotic sister chromatid separation	0.00	0.00
GO:0032103	positive regulation of response to external stimulus	0.00	0.00
GO:0007080	mitotic metaphase plate congression	0.00	0.00
GO:0009410	response to xenobiotic stimulus	0.00	0.00
GO:0001570	vasculogenesis	0.00	0.00
GO:0050900	leukocyte migration	0.00	0.00
GO:0051983	regulation of chromosome segregation	0.00	0.00
GO:0010810	regulation of cell-substrate adhesion	0.00	0.00
GO:0051303	establishment of chromosome localization	0.00	0.00
GO:0051984	positive regulation of chromosome segregation	0.00	0.00
GO:0033045	regulation of sister chromatid segregation	0.00	0.00
GO:0003007	heart morphogenesis	0.00	0.00
GO:0042060	wound healing	0.00	0.00
GO:0051304	chromosome separation	0.00	0.00
GO:0030856	regulation of epithelial cell differentiation	0.00	0.00
GO:0045785	positive regulation of cell adhesion	0.00	0.00
GO:0010466	negative regulation of peptidase activity	0.00	0.00
GO:0008608	attachment of spindle microtubules to kinetochore	0.00	0.00
GO:0019731	antibacterial humoral response	0.00	0.00
GO:1905818	regulation of chromosome separation	0.00	0.00
GO:0003158	endothelium development	0.00	0.00
GO:0043393	regulation of protein binding	0.00	0.00
GO:0043410	positive regulation of MAPK cascade	0.00	0.00
GO:0003018	vascular process in circulatory system	0.00	0.00
GO:0050000	chromosome localization	0.00	0.00
GO:0051783	regulation of nuclear division	0.00	0.00
GO:0045601	regulation of endothelial cell differentiation	0.00	0.00
GO:0044703	multi-organism reproductive process	0.00	0.00
GO:0052548	regulation of endopeptidase activity	0.00	0.00

GO:0002673	regulation of acute inflammatory response	0.00	0.00
GO:0009636	response to toxic substance	0.00	0.00
GO:0044706	multi-multicellular organism process	0.00	0.00
GO:0140014	mitotic nuclear division	0.00	0.00
GO:0045123	cellular extravasation	0.00	0.00
GO:0007565	female pregnancy	0.00	0.00
GO:0006898	receptor-mediated endocytosis	0.00	0.00
GO:0006957	complement activation, alternative pathway	0.00	0.00
GO:0051988	regulation of attachment of spindle microtubules to kinetochore	0.00	0.00
GO:1901970	positive regulation of mitotic sister chromatid separation	0.00	0.00
GO:0007596	blood coagulation	0.00	0.00
GO:1990266	neutrophil migration	0.00	0.00
GO:0007159	leukocyte cell-cell adhesion	0.00	0.00
GO:0006837	serotonin transport	0.00	0.00
GO:0002446	neutrophil mediated immunity	0.00	0.00
GO:1904645	response to amyloid-beta	0.00	0.00
GO:0050817	coagulation	0.00	0.00
GO:0007599	hemostasis	0.00	0.00
GO:0001558	regulation of cell growth	0.00	0.00
GO:0061756	leukocyte adhesion to vascular endothelial cell	0.00	0.00
GO:0031349	positive regulation of defense response	0.00	0.00
GO:2000116	regulation of cysteine-type endopeptidase activity	0.00	0.00
GO:0002274	myeloid leukocyte activation	0.00	0.01
GO:0045765	regulation of angiogenesis	0.00	0.01
GO:0034329	cell junction assembly	0.00	0.01
GO:0000070	mitotic sister chromatid segregation	0.00	0.01
GO:1901888	regulation of cell junction assembly	0.00	0.01
GO:0000819	sister chromatid segregation	0.00	0.01
GO:0045861	negative regulation of proteolysis	0.00	0.01
GO:0043281	regulation of cysteine-type endopeptidase activity involved in apoptotic process	0.00	0.01
GO:0030595	leukocyte chemotaxis	0.00	0.01
GO:0070555	response to interleukin-1	0.00	0.01
GO:1901342	regulation of vasculature development	0.00	0.01
GO:0072001	renal system development	0.00	0.01
GO:0071404	cellular response to low-density lipoprotein particle stimulus	0.00	0.01
GO:0072574	hepatocyte proliferation	0.00	0.01
GO:0072575	epithelial cell proliferation involved in liver morphogenesis	0.00	0.01
GO:0000280	nuclear division	0.00	0.01
GO:0002526	acute inflammatory response	0.00	0.01
GO:0050680	negative regulation of epithelial cell proliferation	0.00	0.01
GO:0098742	cell-cell adhesion via plasma-membrane adhesion molecules	0.00	0.01
GO:1990748	cellular detoxification	0.00	0.01
GO:0032731	positive regulation of interleukin-1 beta production	0.00	0.01

GO:0045453	bone resorption	0.00	0.01
GO:0042119	neutrophil activation	0.00	0.01
GO:0045124	regulation of bone resorption	0.00	0.01
GO:0048143	astrocyte activation	0.00	0.01
GO:0072576	liver morphogenesis	0.00	0.01
GO:0046849	bone remodeling	0.00	0.01
GO:0050804	modulation of chemical synaptic transmission	0.00	0.01
GO:0050830	defense response to Gram-positive bacterium	0.00	0.01
GO:1902412	regulation of mitotic cytokinesis	0.00	0.01
GO:0099177	regulation of trans-synaptic signaling	0.00	0.01
GO:0043270	positive regulation of ion transport	0.00	0.01
GO:0010951	negative regulation of endopeptidase activity	0.00	0.01
GO:0014002	astrocyte development	0.00	0.01
GO:0016049	cell growth	0.00	0.01
GO:0001885	endothelial cell development	0.00	0.01
GO:2000117	negative regulation of cysteine-type endopeptidase activity	0.00	0.01
GO:1901653	cellular response to peptide	0.00	0.01
GO:0098754	detoxification	0.00	0.01
GO:0097237	cellular response to toxic substance	0.00	0.01
GO:0048251	elastic fiber assembly	0.00	0.01
GO:0051256	mitotic spindle midzone assembly	0.00	0.01
GO:0051610	serotonin uptake	0.00	0.01
GO:1901731	positive regulation of platelet aggregation	0.00	0.01
GO:0001933	negative regulation of protein phosphorylation	0.00	0.01
GO:0034109	homotypic cell-cell adhesion	0.00	0.01
GO:0010811	positive regulation of cell-substrate adhesion	0.00	0.01
GO:0019730	antimicrobial humoral response	0.00	0.01
GO:0060343	trabecula formation	0.00	0.01
GO:0097530	granulocyte migration	0.00	0.01
GO:0085029	extracellular matrix assembly	0.00	0.01
GO:0051047	positive regulation of secretion	0.00	0.01
GO:1901991	negative regulation of mitotic cell cycle phase transition	0.00	0.01
GO:0046683	response to organophosphorus	0.00	0.01
GO:0070527	platelet aggregation	0.00	0.01
GO:0036230	granulocyte activation	0.00	0.01
GO:0048286	lung alveolus development	0.00	0.01
GO:0061383	trabecula morphogenesis	0.00	0.01
GO:1905820	positive regulation of chromosome separation	0.00	0.01
GO:0001503	ossification	0.00	0.01
GO:0000022	mitotic spindle elongation	0.00	0.01
GO:0001867	complement activation, lectin pathway	0.00	0.01
GO:0010454	negative regulation of cell fate commitment	0.00	0.01
GO:0090232	positive regulation of spindle checkpoint	0.00	0.01

GO:0090267	positive regulation of mitotic cell cycle spindle assembly checkpoint	0.00	0.01
GO:0044772	mitotic cell cycle phase transition	0.00	0.01
GO:1900046	regulation of hemostasis	0.00	0.01
GO:0001822	kidney development	0.00	0.01
GO:0046850	regulation of bone remodeling	0.00	0.01
GO:0061028	establishment of endothelial barrier	0.00	0.01
GO:1901976	regulation of cell cycle checkpoint	0.00	0.01
GO:0098869	cellular oxidant detoxification	0.00	0.01
GO:0071560	cellular response to transforming growth factor beta stimulus	0.00	0.01
GO:0006979	response to oxidative stress	0.00	0.01
GO:0150076	neuroinflammatory response	0.00	0.01
GO:0002675	positive regulation of acute inflammatory response	0.00	0.01
GO:0030194	positive regulation of blood coagulation	0.00	0.01
GO:1900048	positive regulation of hemostasis	0.00	0.01
GO:0015908	fatty acid transport	0.00	0.01
GO:0043114	regulation of vascular permeability	0.00	0.01
GO:0006956	complement activation	0.00	0.01
GO:0007585	respiratory gaseous exchange by respiratory system	0.00	0.01
GO:0032732	positive regulation of interleukin-1 production	0.00	0.01
GO:1901990	regulation of mitotic cell cycle phase transition	0.00	0.01
GO:0030168	platelet activation	0.00	0.01
GO:0070372	regulation of ERK1 and ERK2 cascade	0.00	0.01
GO:0032494	response to peptidoglycan	0.00	0.01
GO:0070486	leukocyte aggregation	0.00	0.01
GO:0090235	regulation of metaphase plate congression	0.00	0.01
GO:0072012	glomerulus vasculature development	0.00	0.01
GO:0071559	response to transforming growth factor beta	0.00	0.01
GO:0048732	gland development	0.00	0.01
GO:1903532	positive regulation of secretion by cell	0.00	0.01
GO:0000302	response to reactive oxygen species	0.00	0.01
GO:0042445	hormone metabolic process	0.00	0.01
GO:0060326	cell chemotaxis	0.00	0.01
GO:0001655	urogenital system development	0.00	0.01
GO:0042744	hydrogen peroxide catabolic process	0.00	0.01
GO:0050820	positive regulation of coagulation	0.00	0.01
GO:0061041	regulation of wound healing	0.00	0.01
GO:0071674	mononuclear cell migration	0.00	0.01
GO:0030593	neutrophil chemotaxis	0.00	0.01
GO:0010543	regulation of platelet activation	0.00	0.01
GO:0045444	fat cell differentiation	0.00	0.01
GO:0030879	mammary gland development	0.00	0.01
GO:0043154	negative regulation of cysteine-type endopeptidase activity involved in apoptotic process	0.00	0.01

GO:1903034	regulation of response to wounding	0.00	0.01
GO:0002444	myeloid leukocyte mediated immunity	0.00	0.01
GO:0032963	collagen metabolic process	0.00	0.01
GO:0051231	spindle elongation	0.00	0.01
GO:0051255	spindle midzone assembly	0.00	0.01
GO:0045216	cell-cell junction organization	0.00	0.01
GO:0001945	lymph vessel development	0.00	0.01
GO:0061437	renal system vasculature development	0.00	0.01
GO:0061440	kidney vasculature development	0.00	0.01
GO:1900745	positive regulation of p38MAPK cascade	0.00	0.01
GO:0007093	mitotic cell cycle checkpoint signaling	0.00	0.01
GO:0060541	respiratory system development	0.00	0.01
GO:0061900	glial cell activation	0.00	0.01
GO:0061384	heart trabecula morphogenesis	0.00	0.01
GO:0014074	response to purine-containing compound	0.00	0.01
GO:0016485	protein processing	0.00	0.01
GO:0045862	positive regulation of proteolysis	0.00	0.01
GO:0051098	regulation of binding	0.00	0.01
GO:0032611	interleukin-1 beta production	0.00	0.01
GO:0032651	regulation of interleukin-1 beta production	0.00	0.01
GO:0071347	cellular response to interleukin-1	0.00	0.01
GO:0042743	hydrogen peroxide metabolic process	0.00	0.01
GO:0014857	regulation of skeletal muscle cell proliferation	0.00	0.01
GO:0060347	heart trabecula formation	0.00	0.01
GO:0070207	protein homotrimerization	0.00	0.01
GO:2000345	regulation of hepatocyte proliferation	0.00	0.01
GO:0051346	negative regulation of hydrolase activity	0.00	0.01
GO:0033044	regulation of chromosome organization	0.00	0.01
GO:0048771	tissue remodeling	0.00	0.02
GO:0050901	leukocyte tethering or rolling	0.00	0.02
GO:0006939	smooth muscle contraction	0.00	0.02
GO:1905954	positive regulation of lipid localization	0.00	0.02
GO:0060562	epithelial tube morphogenesis	0.00	0.02
GO:0042180	cellular ketone metabolic process	0.00	0.02
GO:0015844	monoamine transport	0.00	0.02
GO:0055094	response to lipoprotein particle	0.00	0.02
GO:0018108	peptidyl-tyrosine phosphorylation	0.00	0.02
GO:0090287	regulation of cellular response to growth factor stimulus	0.00	0.02
GO:0015671	oxygen transport	0.00	0.02
GO:0034112	positive regulation of homotypic cell-cell adhesion	0.00	0.02
GO:0060841	venous blood vessel development	0.00	0.02
GO:1900747	negative regulation of vascular endothelial growth factor signaling pathway	0.00	0.02
GO:0048640	negative regulation of developmental growth	0.00	0.02

GO:0042326	negative regulation of phosphorylation	0.00	0.02
GO:0018212	peptidyl-tyrosine modification	0.00	0.02
GO:0042742	defense response to bacterium	0.00	0.02
GO:0070371	ERK1 and ERK2 cascade	0.00	0.02
GO:0060828	regulation of canonical Wnt signaling pathway	0.00	0.02
GO:0070374	positive regulation of ERK1 and ERK2 cascade	0.00	0.02
GO:0043903	regulation of biological process involved in symbiotic interaction	0.00	0.02
GO:0019915	lipid storage	0.00	0.02
GO:0050679	positive regulation of epithelial cell proliferation	0.00	0.02
GO:0030324	lung development	0.00	0.02
GO:0007043	cell-cell junction assembly	0.00	0.02
GO:0007096	regulation of exit from mitosis	0.00	0.02
GO:0008228	opsonization	0.00	0.02
GO:0070206	protein trimerization	0.00	0.02
GO:0072189	ureter development	0.00	0.02
GO:1901550	regulation of endothelial cell development	0.00	0.02
GO:1903140	regulation of establishment of endothelial barrier	0.00	0.02
GO:0016486	peptide hormone processing	0.00	0.02
GO:0071711	basement membrane organization	0.00	0.02
GO:0030308	negative regulation of cell growth	0.00	0.02
GO:0007178	transmembrane receptor protein serine/threonine kinase signaling pathway	0.00	0.02
GO:0071375	cellular response to peptide hormone stimulus	0.00	0.02
GO:0050730	regulation of peptidyl-tyrosine phosphorylation	0.00	0.02
GO:0050729	positive regulation of inflammatory response	0.00	0.02
GO:0046456	icosanoid biosynthetic process	0.00	0.02
GO:0032102	negative regulation of response to external stimulus	0.00	0.02
GO:0007162	negative regulation of cell adhesion	0.00	0.02
GO:0071402	cellular response to lipoprotein particle stimulus	0.00	0.02
GO:0031623	receptor internalization	0.00	0.02
GO:0032355	response to estradiol	0.00	0.02
GO:0090100	positive regulation of transmembrane receptor protein serine/threonine kinase signaling pathway	0.00	0.02
GO:0030323	respiratory tube development	0.00	0.02
GO:0051591	response to cAMP	0.00	0.02
GO:0010876	lipid localization	0.00	0.02
GO:0032868	response to insulin	0.00	0.02
GO:0071900	regulation of protein serine/threonine kinase activity	0.00	0.02
GO:0090303	positive regulation of wound healing	0.00	0.02
GO:0008037	cell recognition	0.00	0.02
GO:0010755	regulation of plasminogen activation	0.00	0.02
GO:0016264	gap junction assembly	0.00	0.02
GO:0034501	protein localization to kinetochore	0.00	0.02
GO:0043116	negative regulation of vascular permeability	0.00	0.02

GO:0051917	regulation of fibrinolysis	0.00	0.02
GO:1902548	negative regulation of cellular response to vascular endothelial growth factor stimulus	0.00	0.02
GO:1903083	protein localization to condensed chromosome	0.00	0.02
GO:2000811	negative regulation of anoikis	0.00	0.02
GO:0140448	signaling receptor ligand precursor processing	0.00	0.02
GO:0010952	positive regulation of peptidase activity	0.00	0.02
GO:0032757	positive regulation of interleukin-8 production	0.00	0.02
GO:0060485	mesenchyme development	0.00	0.02
GO:0007051	spindle organization	0.00	0.02
GO:0006631	fatty acid metabolic process	0.00	0.02
GO:0001952	regulation of cell-matrix adhesion	0.00	0.02
GO:0002283	neutrophil activation involved in immune response	0.00	0.02
GO:0010885	regulation of cholesterol storage	0.00	0.02
GO:1901978	positive regulation of cell cycle checkpoint	0.00	0.02
GO:0003206	cardiac chamber morphogenesis	0.00	0.02
GO:0010038	response to metal ion	0.00	0.02
GO:1903035	negative regulation of response to wounding	0.00	0.02
GO:0071248	cellular response to metal ion	0.00	0.02
GO:0006940	regulation of smooth muscle contraction	0.00	0.02
GO:0031100	animal organ regeneration	0.00	0.02
GO:0033674	positive regulation of kinase activity	0.00	0.02
GO:0006882	cellular zinc ion homeostasis	0.00	0.02
GO:0007160	cell-matrix adhesion	0.00	0.02
GO:0051225	spindle assembly	0.00	0.02
GO:0060389	pathway-restricted SMAD protein phosphorylation	0.00	0.02
GO:0098813	nuclear chromosome segregation	0.00	0.02
GO:0019217	regulation of fatty acid metabolic process	0.00	0.02
GO:0007171	activation of transmembrane receptor protein tyrosine kinase activity	0.00	0.03
GO:0014856	skeletal muscle cell proliferation	0.00	0.03
GO:0048670	regulation of collateral sprouting	0.00	0.03
GO:0060749	mammary gland alveolus development	0.00	0.03
GO:0061377	mammary gland lobule development	0.00	0.03
GO:0002691	regulation of cellular extravasation	0.00	0.03
GO:0015909	long-chain fatty acid transport	0.00	0.03
GO:0032612	interleukin-1 production	0.00	0.03
GO:0032652	regulation of interleukin-1 production	0.00	0.03
GO:0071621	granulocyte chemotaxis	0.00	0.03
GO:0048661	positive regulation of smooth muscle cell proliferation	0.00	0.03
GO:0003205	cardiac chamber development	0.00	0.03
GO:0030509	BMP signaling pathway	0.00	0.03
GO:0010453	regulation of cell fate commitment	0.00	0.03
GO:0055069	zinc ion homeostasis	0.00	0.03

GO:0032755	positive regulation of interleukin-6 production	0.00	0.03
GO:0030858	positive regulation of epithelial cell differentiation	0.00	0.03
GO:0010878	cholesterol storage	0.00	0.03
GO:0060353	regulation of cell adhesion molecule production	0.00	0.03
GO:0007052	mitotic spindle organization	0.00	0.03
GO:0045930	negative regulation of mitotic cell cycle	0.00	0.03
GO:0002251	organ or tissue specific immune response	0.00	0.03
GO:0007595	lactation	0.00	0.03
GO:0033002	muscle cell proliferation	0.00	0.03
GO:0090130	tissue migration	0.00	0.03
GO:0030193	regulation of blood coagulation	0.00	0.03
GO:0007156	homophilic cell adhesion via plasma membrane adhesion molecules	0.00	0.03
GO:0050806	positive regulation of synaptic transmission	0.00	0.03
GO:0045860	positive regulation of protein kinase activity	0.00	0.03
GO:0048638	regulation of developmental growth	0.00	0.03
GO:0030100	regulation of endocytosis	0.00	0.03
GO:0090092	regulation of transmembrane receptor protein serine/threonine kinase signaling pathway	0.00	0.03
GO:0002548	monocyte chemotaxis	0.00	0.03
GO:0060537	muscle tissue development	0.00	0.03
GO:0035313	wound healing, spreading of epidermal cells	0.00	0.03
GO:0010565	regulation of cellular ketone metabolic process	0.00	0.03
GO:0015850	organic hydroxy compound transport	0.00	0.03
GO:0016042	lipid catabolic process	0.00	0.03
GO:0030111	regulation of Wnt signaling pathway	0.00	0.03
GO:0031214	biomineral tissue development	0.00	0.03
GO:0070482	response to oxygen levels	0.00	0.03
GO:0030574	collagen catabolic process	0.00	0.03
GO:1900744	regulation of p38MAPK cascade	0.00	0.03
GO:0002064	epithelial cell development	0.00	0.03
GO:0006790	sulfur compound metabolic process	0.00	0.03
GO:0035493	SNARE complex assembly	0.00	0.03
GO:0022900	electron transport chain	0.00	0.03
GO:0060538	skeletal muscle organ development	0.00	0.03
GO:0042058	regulation of epidermal growth factor receptor signaling pathway	0.00	0.03
GO:0050766	positive regulation of phagocytosis	0.00	0.03
GO:0090307	mitotic spindle assembly	0.00	0.03
GO:0006469	negative regulation of protein kinase activity	0.00	0.03
GO:0007179	transforming growth factor beta receptor signaling pathway	0.00	0.03
GO:0071772	response to BMP	0.00	0.04
GO:0071773	cellular response to BMP stimulus	0.00	0.04
GO:0006869	lipid transport	0.00	0.04
GO:0050818	regulation of coagulation	0.00	0.04

GO:0001774	microglial cell activation	0.00	0.04
GO:0051180	vitamin transport	0.00	0.04
GO:0072330	monocarboxylic acid biosynthetic process	0.00	0.04
GO:0036303	lymph vessel morphogenesis	0.00	0.04
GO:0051043	regulation of membrane protein ectodomain proteolysis	0.00	0.04
GO:0060352	cell adhesion molecule production	0.00	0.04
GO:0033138	positive regulation of peptidyl-serine phosphorylation	0.01	0.04
GO:0090263	positive regulation of canonical Wnt signaling pathway	0.01	0.04
GO:0022411	cellular component disassembly	0.01	0.04
GO:0110148	biomineralization	0.01	0.04
GO:0062023	collagen-containing extracellular matrix	0.00	0.00
GO:0005581	collagen trimer	0.00	0.00
GO:0045121	membrane raft	0.00	0.00
GO:0098857	membrane microdomain	0.00	0.00
GO:0030139	endocytic vesicle	0.00	0.00
GO:0009897	external side of plasma membrane	0.00	0.00
GO:0005791	rough endoplasmic reticulum	0.00	0.00
GO:0031092	platelet alpha granule membrane	0.00	0.00
GO:0042599	lamellar body	0.00	0.00
GO:0042581	specific granule	0.00	0.00
GO:0031225	anchored component of membrane	0.00	0.00
GO:0030667	secretory granule membrane	0.00	0.00
GO:0045177	apical part of cell	0.00	0.01
GO:0034774	secretory granule lumen	0.00	0.01
GO:0060205	cytoplasmic vesicle lumen	0.00	0.01
GO:0031983	vesicle lumen	0.00	0.01
GO:0045334	clathrin-coated endocytic vesicle	0.00	0.01
GO:0016324	apical plasma membrane	0.00	0.01
GO:0031838	haptoglobin-hemoglobin complex	0.00	0.01
GO:1905370	serine-type endopeptidase complex	0.00	0.01
GO:0005833	hemoglobin complex	0.00	0.01
GO:0070820	tertiary granule	0.00	0.01
GO:1905286	serine-type peptidase complex	0.00	0.01
GO:0005796	Golgi lumen	0.00	0.01
GO:1904724	tertiary granule lumen	0.00	0.01
GO:0005901	caveola	0.00	0.01
GO:0044853	plasma membrane raft	0.00	0.01
GO:0035580	specific granule lumen	0.00	0.02
GO:0031091	platelet alpha granule	0.00	0.02
GO:0042827	platelet dense granule	0.00	0.03
GO:0000775	chromosome, centromeric region	0.00	0.03
GO:0042383	sarcolemma	0.00	0.03
GO:0044291	cell-cell contact zone	0.00	0.03
GO:0071682	endocytic vesicle lumen	0.00	0.04

GO:0005201	extracellular matrix structural constituent	0.00	0.00
GO:0005539	glycosaminoglycan binding	0.00	0.00
GO:0038024	cargo receptor activity	0.00	0.00
GO:0061134	peptidase regulator activity	0.00	0.00
GO:0008201	heparin binding	0.00	0.00
GO:0005044	scavenger receptor activity	0.00	0.00
GO:0005518	collagen binding	0.00	0.00
GO:0030246	carbohydrate binding	0.00	0.00
GO:0017171	serine hydrolase activity	0.00	0.01
GO:0031406	carboxylic acid binding	0.00	0.01
GO:0001540	amyloid-beta binding	0.00	0.01
GO:0004252	serine-type endopeptidase activity	0.00	0.01
GO:0031720	haptoglobin binding	0.00	0.01
GO:0098631	cell adhesion mediator activity	0.00	0.01
GO:0008237	metallopeptidase activity	0.00	0.02
GO:0030414	peptidase inhibitor activity	0.00	0.02
GO:0005178	integrin binding	0.00	0.02
GO:0008236	serine-type peptidase activity	0.00	0.02
GO:0005504	fatty acid binding	0.00	0.02
GO:1901681	sulfur compound binding	0.00	0.02
GO:0004089	carbonate dehydratase activity	0.00	0.02
GO:0005344	oxygen carrier activity	0.00	0.02
GO:0043177	organic acid binding	0.00	0.02
GO:0098632	cell-cell adhesion mediator activity	0.00	0.02
GO:0036041	long-chain fatty acid binding	0.00	0.02
GO:0070492	oligosaccharide binding	0.00	0.02
GO:0004601	peroxidase activity	0.00	0.02
GO:0033293	monocarboxylic acid binding	0.00	0.02
GO:0048306	calcium-dependent protein binding	0.00	0.02
GO:0050840	extracellular matrix binding	0.00	0.02
GO:0004222	metalloendopeptidase activity	0.00	0.02
GO:0016684	oxidoreductase activity, acting on peroxide as acceptor	0.00	0.02
GO:0005324	long-chain fatty acid transporter activity	0.00	0.03
GO:0050660	flavin adenine dinucleotide binding	0.00	0.03
GO:0004675	transmembrane receptor protein serine/threonine kinase activity	0.00	0.03
GO:0004745	NAD-retinol dehydrogenase activity	0.00	0.03
GO:0019825	oxygen binding	0.00	0.03
GO:0004857	enzyme inhibitor activity	0.00	0.04
GO:0005125	cytokine activity	0.00	0.04
GO:0048018	receptor ligand activity	0.00	0.04

Abbreviation: p-value: Probability; q-value: Adjusted P-value.

Table S5 KEGG pathway enrichment analysis results.

ID	Description	p-value	q-value
hsa05144	Malaria	0.00	0.00
hsa04610	Complement and coagulation cascades	0.00	0.03
hsa00982	Drug metabolism - cytochrome P450	0.00	0.03
hsa00350	Tyrosine metabolism	0.00	0.03
hsa05418	Fluid shear stress and atherosclerosis	0.00	0.05
hsa04512	ECM-receptor interaction	0.00	0.05
hsa04670	Leukocyte transendothelial migration	0.00	0.05
hsa05323	Rheumatoid arthritis	0.00	0.05
hsa04657	IL-17 signaling pathway	0.00	0.05
hsa04145	Phagosome	0.00	0.06
hsa03320	PPAR signaling pathway	0.00	0.06
hsa00430	Taurine and hypotaurine metabolism	0.00	0.06
hsa00910	Nitrogen metabolism	0.00	0.07
hsa05143	African trypanosomiasis	0.01	0.08
hsa05205	Proteoglycans in cancer	0.01	0.09
hsa05417	Lipid and atherosclerosis	0.01	0.12
hsa05150	Staphylococcus aureus infection	0.01	0.12
hsa04110	Cell cycle	0.01	0.12
hsa05410	Hypertrophic cardiomyopathy	0.01	0.12
hsa04514	Cell adhesion molecules	0.01	0.12
hsa05146	Amoebiasis	0.01	0.14
hsa01523	Antifolate resistance	0.02	0.19
hsa05134	Legionellosis	0.02	0.21
hsa04613	Neutrophil extracellular trap formation	0.03	0.28
hsa05171	Coronavirus disease - COVID-19	0.03	0.30
hsa00360	Phenylalanine metabolism	0.04	0.30
hsa00830	Retinol metabolism	0.04	0.30
hsa04664	Fc epsilon RI signaling pathway	0.04	0.30
hsa05415	Diabetic cardiomyopathy	0.04	0.30
hsa04061	Viral protein interaction with cytokine and cytokine receptor	0.04	0.30
hsa04933	AGE-RAGE signaling pathway in diabetic complications	0.04	0.30
hsa05219	Bladder cancer	0.04	0.30
hsa00380	Tryptophan metabolism	0.05	0.30
hsa04974	Protein digestion and absorption	0.05	0.30
hsa05414	Dilated cardiomyopathy	0.05	0.30
hsa00071	Fatty acid degradation	0.05	0.30

Abbreviation: p-value: Probability: q-value: Adjusted P-value.

Table S6 Exposure factors screened by pathway enrichment analysis.

Trait	Consortium	Year	Race	Participants	Number of SNPs	GWAS ID
Coronary atherosclerosis	NA	2021	European	211,203	16,380,447	finn-b-I9_CORATHER
Coronary atherosclerosis	NA	2018	European	361,194	13,586,589	ukb-d-I9_CORATHER
Cell adhesion molecule 3	NA	2018	European	3,301	10,534,735	prot-a-338
Dipeptidase 1	NA	2018	European	3,301	10,534,735	prot-a-856
Thimet oligopeptidase	NA	2020	European	1,313	18,197,903	ebi-a-GCST90010352
Dipeptidyl peptidase 2	NA	2020	European	1,290	18,139,686	ebi-a-GCST90010307

Abbreviation: NA: Not described.

Table S7 Mendelian randomization results for the validation dataset.

Exposure	Method	Beta	OR (95%CI)	P value
Coronary atherosclerosis	MR Egger	-6.22	0.002(1.42E-06-2.76)	0.10
	Weighted median	-3.21	0.40(0.0004-4.05)	0.17
	Inverse variance weighted	-4.05	0.02(0.0007-0.43)	0.01
	Simple mode	0.27	1.32(4.98E-06-3.47E+05)	0.97
	Weighted mode	0.75	2.12(1.33E-05-3.39E+05)	0.90

Abbreviation: P value: Probability； OR: Risk Ratio; 95%CI:95% Confidence Interval.

Table S8 Heterogeneity of the validation dataset and pleiotropic results.

Exposure	Heterogeneity P		MR-Egger regression	
	MR Egger	IVW	Intercept	Intercept P
Coronary atherosclerosis	0.0468	0.0507	0.0089	0.5135

Abbreviation: Heterogeneity P: Heterogeneity P value; IVW: Inverse variance weighted; Intercept P: Intercept P value.
